# PassStat, a simple but fast, precise and versatile open source potentiostat

**DOI:** 10.1016/j.ohx.2022.e00290

**Published:** 2022-03-09

**Authors:** Mélicia Caux, Anis Achit, Kethsovann Var, Gabriel Boitel-Aullen, Daniel Rose, Agnès Aubouy, Sylvain Argentieri, Raymond Campagnolo, Emmanuel Maisonhaute

**Affiliations:** a Sorbonne Université, CNRS, Laboratoire Interfaces et Systèmes Electrochimiques, 4 place Jussieu, 75005 Paris, France; b UMR152 PHARMADEV, Université de Toulouse, IRD, UPS, France; c Sorbonne Université, CNRS, Institut des Systèmes Intelligents et de Robotique, 4 place Jussieu, 75005 Paris, France

**Keywords:** Potentiostat, Cyclic voltammetry, Square wave voltammetry, Ultramicroelectrodes, Open hardware, Analytical chemistry

## Abstract

This work presents 4 open source potentiostat solutions for performing accurate measurements in cyclic voltammetry and square wave voltammetry at a low price. A very simple and easy to reproduce analogic board (c.a. 10 €) was driven either by a Teensy card from the company PJRC under an Arduino/Python software solution (39 €) or by an Analog Discovery 2 device from Digilent (less than 300 €). A smartphone Bluetooth Android interface was also created to circumvent the use of a computer. We demonstrated that our scheme is suitable for measurements in classical electrochemical conditions but also to carry out experiments with ultramicroelectrodes. We could thus reach a noise resolution of less than 1 pA. Scan rates of 8000 Vs^−1^ with ohmic drop compensation were also achieved. The device is suitable for teaching purposes but also for experiments in a participative science context on the ground, or countries with lower financial possibilities.

## Graphical abstract

**Figure:**
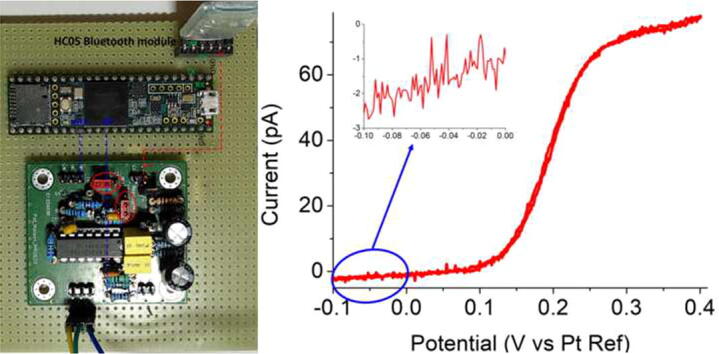

**Specifications table:** 

Hardware name	*PassStat*
Subject area	• Chemistry and biochemistry Educational tools and open source alternatives to existing infrastructure General
Hardware type	• Measuring physical properties and in-lab sensorsOther: Performing electrochemical measurements (cyclic voltammetry and square wave voltammetry)
Closest commercial analog	There are many commercial potentiostats available (Autolab, PalmSens, Biologic, Origalys, CH Instruments)
Open source license	CERN-OHL-S v2 for the hardware GNU GPL v3 for the software Creative Commons v4 for design files and documentation
Cost of hardware	40–303 € depending on the configuration
Source file repository	*https://zenodo.org/record/5719382#.YZwJ17rjJhE* *https://ohwr.org/project/passstat*
OSHWA certification UID *(OPTIONAL)*	*FR000017*

## Hardware in context

Analytical electrochemistry is a domain in continuous expansion because electrochemical measurements are simple, cheap to implement, and can be realized in a point of care approach [1]. Many analytes are electroactive or at least involved in reactions with electroactive species, so that electrochemistry is relevant for environmental, health or food areas among others. Specific electrode preparation is sometimes necessary to reach ultralow concentrations but in many cases non modified electrodes are already sufficient. Some commercial potentiostats present excellent detection limits and are compact enough to be easily transported. Nevertheless, they are still black boxes that are impossible to be repaired by the end user in case of damage. Their relatively high cost prevent their use in resource-limited or within participative science contexts. Therefore, many interesting cheap home-made and open source systems have been proposed for educational purposes or developing countries [2], [3], [4], [5], [6], [7], [8], [9], [10], [11]. In some cases, some devices such as the one proposed by Matsubara reach characteristics close to commercial systems [11]. The heart of a potentiostat is an analytical device that should handle a three electrode configuration, meaning that no current should flow through the reference electrode (RE) while precisely controlling the potential of the working electrode (WE). For that, several operational amplifiers (OAs) are usually used [12]. Those are however very cheap and compact components. Additionally, a generator to impose electrode potential and an acquisition card or processor to register the data are also mandatory. Some open- source devices specifically develop all these elements, which optimizes compactness. For example Whitesides et al., Leech et al. and Rajendran et al. independently developed potentiostats that can be remotely controlled through wifi with a smartphone [3], [7], [9]. Nevertheless, the benefit in size is at the expense of board simplicity and repairability which may discourage new and not trained users to build their own apparatus. Many DIY devices rely on the well-known Arduino Uno microcontroller. Nevertheless, no digital-analytical converter (DAC) is available in this device so that this function either have to be implemented electronically or thanks to another specific component to accurately control the electrode potential and generate the ramp in cyclic voltammetry for example [4]. To face these issues, the Rodeostat [13] proposed by the company Irodeo (250$) and the SweepStat proposed by Glasscott et al. took benefit from the Teensy 3.2 card provided by PJRC that offer DAC outputs and is compatible with the Arduino environment [14]. Nevertheless, the Rodeostat scheme is based on surface mounted component and thus can only be repaired in a well-equipped electronic laboratory. The Sweepstat proposes a rather simple scheme that takes benefit from use of a quad amplifier but it only works in a two electrode configuration. All open source potentiostats are compared in the recent paper by Matsubara (see section J in SI) [11].

Both to propose a low cost but performant device and for educational and intellectual purposes, we studied how to produce the simplest potentiostat scheme working in three electrode configuration. This led us to propose an easy to understand and cheap (<10€) electronic card from which several configurations can be implemented by simply displacing some switches. The first alternative to run the potentiostat is to use positive and negative power supplies, to apply the potential perturbation with a function generator and to acquire the signal with an oscilloscope. In this case, the potentiostat scheme can be resumed to three OAs, three resistors and two capacitors. In this paper, we took benefit from the device Analog Discovery 2 proposed by Digilent (academic price 294€) that proposes all these features in a single USB driven device. The second and cheapest option stands again on the Teensy 3.2 or 3.6 cards (price 24 or 39 €). The potentiostat power supply is directly provided by the USB voltage available from the Teensy. Since USB port power pins are 0 and 5 V, and that no negative tension is available from the computer, the electronic scheme was conceived to handle this problem. An additional Bluetooth module and an external battery can be added easily for remote control using an Android smartphone. Moreover, we wished to examine and push the performances of our system either towards low current (pA) detection or fast scan (several thousands Vs^−1^) voltammetry that we commonly use in our laboratory. This aspect was not treated in previous papers excepted in the work of Matsubara [11]. Below, we first depict the different possible implementations of the potentiostat. We then present representative results obtained in cyclic voltammetry or square wave voltammetry. We took ferrocene as common reference electroactive entity, and paracetamol as typical example of drug analysis application [15], [16], [17], [18], [19], [20], [21], [22], [23], [24], [25]. We named our potentiostat the PassStat, after the French electrochemical YouTube video series “Le Courant Passe” produced by the Societe Chimique de France to catch student interest [26]. All the electronic schemes and driving softwares are provided in Zenodo repository.

## Hardware description

• Cheap and repairable potentiostat card
• Low noise level down to 1 pA
• Fast scan rates up to 8000 Vs^−1^
• Can be driven by Teensy or Analog Discovery 2 devices

For designing our potentiostats, we took advantage of the TI LMC 6484 quad OA as first explored by Glasscott et al. [14]. This component displays very low input currents at the expense of its bandwidth that is limited to 1 MHz which is less than the one required to reach scan rates above 5 × 10^4^ s^−1^ (this was not the purpose of the present work). Having all amplifiers in a same component greatly simplifies the electronic scheme, particularly regarding the power supplies. In the following, we provide one figure per possible implementation. The printed circuit board measured only 5 × 5 cm^2^. We also propose standard values for the electronic components to probe the system in a “standard configuration” paragraph. We noted, as often observed that measurements are improved when realized in a Faraday cage (mandatory for currents lower than 1µA). Even better results were obtained when the power supply of the laptop computer was disconnected (in that case the Faraday cage should be connected to the ground of the computer). A Faraday cage is however not mandatory for standard measurements with millimetric electrodes. The different schemes are provided by increasing complexity. Each labeled component has the same role in various configurations. Jumpers on the printed circuit board card allow to switch between different configurations. Table 1 summarizes the main characteristics of the different configurations.

**Table 1:** Main characteristics of different PassStat configurations.

Name	Potential Range (V)	Microcontroller	Interface	Maximum scan rate	Remote control	Price (€)
PassStat 1	5 V with AD2, up to 15 V	Analog Discovery 2	Waveforms from Digilent	8000 Vs^−1^	no	310
PassStat 2.0	1.6 V	Teensy 3.6	Arduino/Python	66 Vs^−1^	no	50
PassStat 2.1	2.4 V	Teensy 3.6	Arduino/Python	100 Vs^−1^	no	50
PassStat 2.2	2.4 V	Teensy 3.6	Android Studio	100 Vs^−1^	Bluetooth	70

### PassStat 1.0: Simplest and fastest design.

**Fig. 1:**
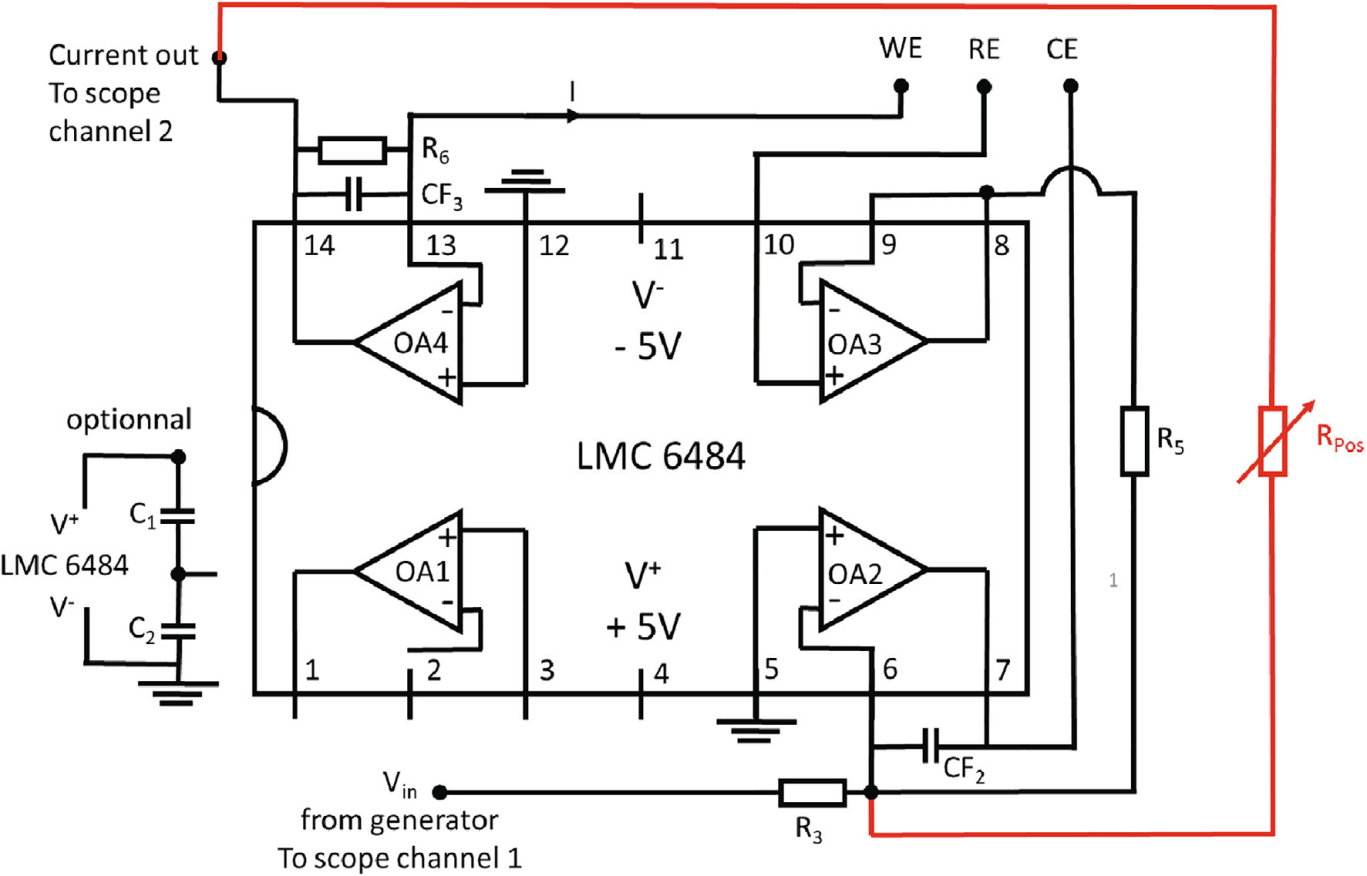
PassStat 1.0 scheme. The R_pos_ potentiometer is optional and devoted to ohmic drop compensation. (For interpretation of the references to colour in this figure legend, the reader is referred to the web version of this article.)

This first setup, which represents the classical scheme [12], [27], [28], [28], [29], [30], is presented in Fig. 1. Here, only three resistors and two capacitors (to stabilize the amplifiers) are used. Amplifier OA3 is used as follower in order to ensure that no current flows through the reference electrode while OA2 adjusts the potential of the counter electrode. The gain of the current-tension converter OA4 can be tuned thanks to resistor R_6_, and capacitor CF_3_ in parallel can be adjusted to filter the noise while keeping R_6_C_6_ low compared to the characteristic timescale of the measurements to keep the signal integrity.

In this work, this setup was implemented with an Analog Discovery 2. This device includes one positive and one negative power supplies (V^+^ = +5V maximum, V^−^=−5V minimum), two signal generators and two scope channels. For a larger compliance supplying up to ± 15 V is possible (but necessitates two additional power supplies). The generator output was sent to the potentiostat to provide the electrode potential (*i.e.* the ramp in cyclic voltammetry). It was also sent to the first scope channel to measure the potential although this is not completely necessary.

This simple setup allows to benefit from the whole bandwidth of the potentiostat. On a test with a resistor, we noted that with CF_2_ = 15 pF, CF_3_ = 3.3 pF and R_6_ = 10 kΩ there is no apparent amplitude diminution at 40,000 Vs^−1^. In these conditions the phase shift translates into a temporal delay between the current and potential of 0.5 µs. Peak potentials would then be altered by 5 mV at 10,000 Vs^−1^. These estimations were however carried out without ohmic drop compensation that is usually necessary at large scan rates and may alter the bandwidth (see below for experimental results on ferrocene).

*Standard configuration:* R_3_ = R_5_ = 10 kΩ. CF_2_ = 15 pF. CF_3_ = 1 nF. R_6_ = 1 MΩ. This configuration is suitable for probing a 1 mM ferrocene solution in acetonitrile with a 0.5 mm diameter platinum electrode at scan rates between 0.05 and 5 Vs^−1^. For larger scan rates or electrode diameters, the current will saturate. Then diminish R_6_ to 100 or 10 kΩ and CF_3_ progressively down to 3 pF for increasing the bandwidth.

Although this configuration is the most powerful and versatile, the final price of the device may be limiting. In addition, programming the generator for cyclic voltammetry and chronoamperometry is not very difficult. For more complicated potential ramps such as those used in square wave voltammetry or differential pulse voltammetry for example, a specific program should be elaborated (see section E in the work of Matsubara) [11]. The subsequent configurations solve these issues by using a low price interface card together with a dedicated software.

### Ohmic drop compensation

Ohmic drop occurs when large electrodes or relatively fast scan rates are used. It is due to the non-negligible electrolyte resistor between the working and reference electrodes. This effect distorts the signal but can be compensated by using small electrodes and adding electronically in real time a tension proportional to the current output to the voltage ramp. Further information about ohmic drop compensation may be found in the litterature [12], [28], [29], [31], [32]. Only PassStat 1.0 configuration can integrate ohmic drop compensation. For that, a potentiometer should be added between pins 14 and 6 as shown by the red addons in Fig. 1. The potentiometer value should take into account the solution resistor (see analytical calculations below). When ohmic drop compensation gets close to 100%, oscillations appear at the initial and inversion potentials, as explained in refs [27], [28], [29], [30], [31], [32].

### PassStat 2: Plug and play low cost configuration with a Teensy card

In the following several configurations of the potentiostat are available for driving with a Teensy 3.2 or 3.6 card. These cards provide the advantage of having one (3.2) or 2 (3.6) analog outputs that can be used for defining accurately the electrode potential. They work with the Arduino IDE after installation of the Teensyduino add-on. A Python software with a graphical interface was also programmed to send the parameters (for example number of cycles, scan rate, potential excursion in CV) and collect the data. However, the potentiostat scheme needs to be adapted because the Teensy card does not provide V^+^ and V^−^ power supplies unlike the analog discovery. There are however a 5 V output (in fact the tension provided by the USB supply that stands near 5 V) and a 3.3 V one. To face this problem of dissymmetrical tensions, we added on the board voltage dividers to produce intermediate voltages for the positive input E^+^ of the amplifiers OA1, OA2 and OA4 that are thus not any more connected to the ground. Capacitors C_1_ = C_2_ = 47 µF and C_3_ = C_4_ = 1 µF damp the possible fluctuations of the power supply. In our conditions, they could be removed without alteration of the data but we consider that it is safer to include them. V^−^ is then connected to ground and V^+^ to 3.3 (PassStat 2.0) or 5 V (PassStat 2.1 and 2.2). We show below that the electrode potential, thus V_W_ – V_RE_ can be accurately controlled by this way.

The Teensy analog output is 12 bits over a 3.3 V range so that each potential increment is 0.8 mV (PassStat 2.0) or 1.2 mV (PassStat 2.1 and 2.2). Such values are precise enough for most electrochemical experiments. The input resolution for the current can be chosen up to 16 bits. The Teensy card allows applying and measuring data as fast as 12 µs. This limits the scan rate to 66 Vs^−1^ for PassStat 2.0 and 100 Vs^−1^ for PassStat 2.1 and 2.2. Faster scan rate could be achieved using potential steps larger than 0.8 or 1.2 mV but this limit is sufficient for most analytical electrochemistry experiments. Since USB voltage is not precisely 5.00 V on all computers, a calibration procedure, detailed below, is necessary if accurate measurements are desired. A precise voltage reference stage can also be used in case voltage fluctuations are observed but this was found not necessary in our case.

#### PassStat 2.0: Simple but compliance limited to 1.6 V

**Fig. 2:**
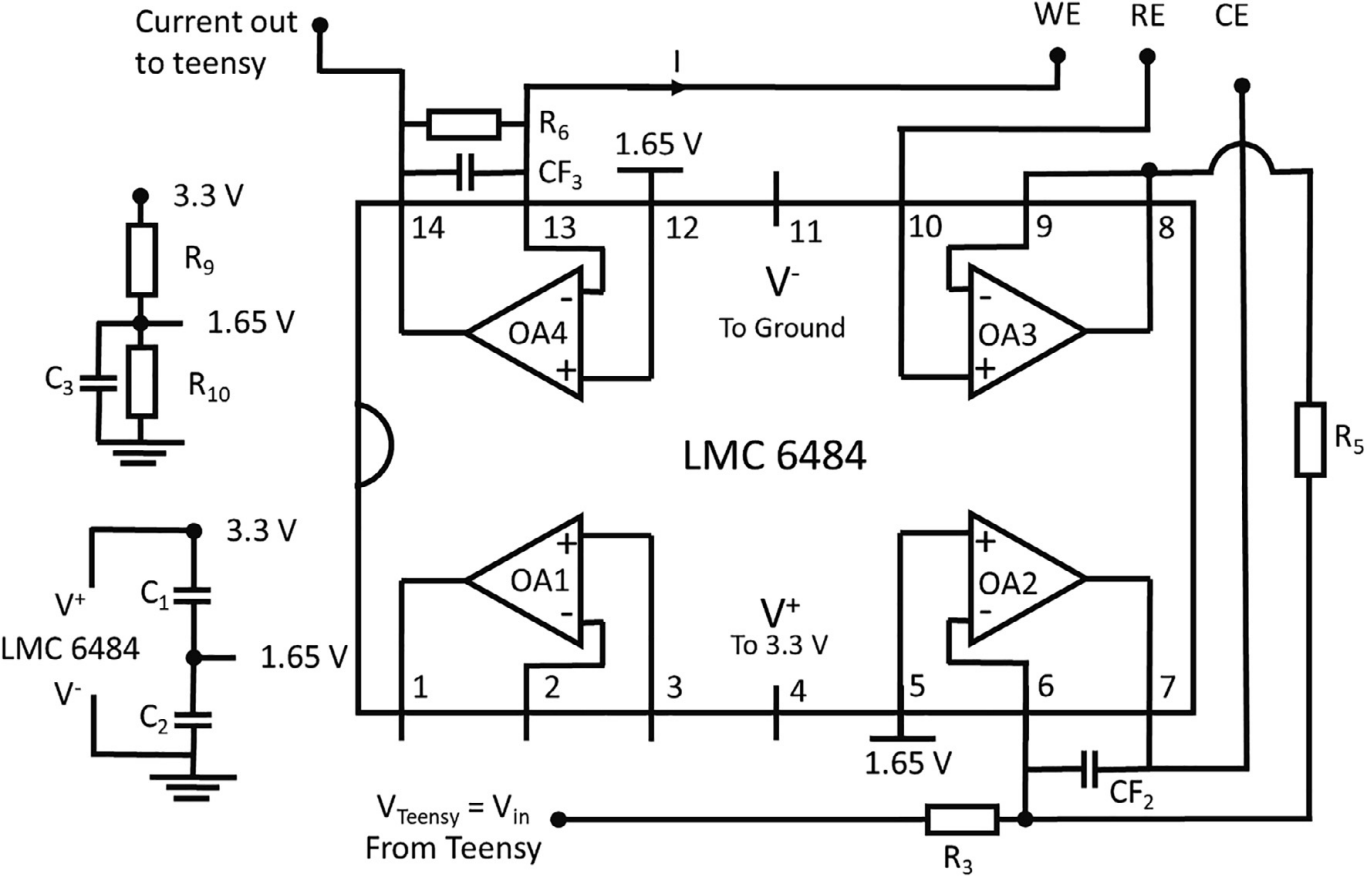
PassStat 2.0 scheme.

Here, the 3.3 V output is connected to V^+^. V^−^ is connected to the analog ground as displayed in Fig. 2. Resistors R_9_ and R_10_ (1 kΩ) are used to provide a 1.65 V tension for the E^+^ entry of OA2 and OA4. This configuration may be used when the best current and potential precisions are desired (0.8 mV on the DAC and ADC instead of 1.2 mV). It is for example suitable with ultramicroelectrodes but may be limited if the potential excursion should be extended beyond ± 1.6 V or for large electrodes for which the counter electrode needs a larger compliance.

*Standard configuration:* R_9_ = R_10_ = 1 kΩ. R_3_ = R_5_ = 10 kΩ. CF_2_ = CF_3_ = 100 pF. R_6_ = 100 MΩ. This configuration is suitable for probing a 1 mM ferrocene solution in acetonitrile with a 12.5 µm radius electrode at 10 mVs^−1^. A steady state CV should be observed. For smaller electrodes or concentrations, increase R_6_ up to 1 GΩ.

#### PassStat 2.1: Extended compliance to 2.4 V

In order to benefit from the maximum compliance possible with the Teensy card, V^+^ is now connected to the V^in^ pin of the Teensy card that is powered directly by the USB lead (an external battery could also be used). The analog output range is extended thanks to an inverting voltage amplifier stage implemented with OA1, the first amplifier of LMC6484 with resistors R_1_ (10 kΩ) and R_2_ (15 kΩ). Calculations detailed below show that E^+^ of this OA should be poised to 2 V, thus another voltage divider was implemented thanks to resistors R_11_ (1.5 kΩ) and R_12_ (1 kΩ). The voltage precision on the DAC and ADC is now thus 0.8x1.5 = 1.2 mV.

**Fig. 3:**
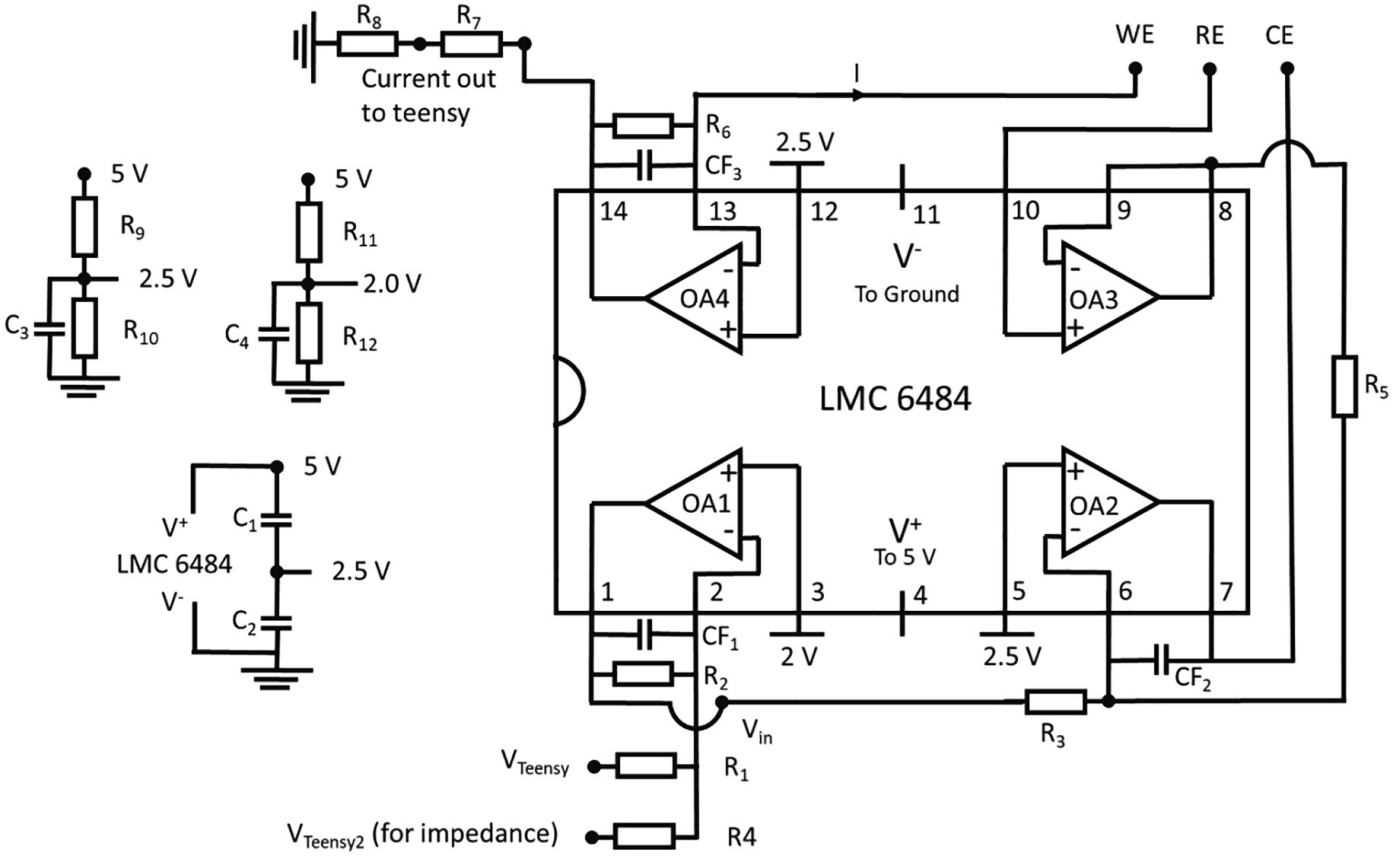
PassStat 2.1 scheme.

Nevertheless, tension at the analog inputs of the Teensy should not be above 3.3 V to avoid irreversible damage to the card. To face this issue, voltage at the output of the current-tension converter (V_14_, OA4) is converted back to a 0–3.3 V range thanks to another voltage divider (resistors R_7_ = 510 Ω and R_8_ = 1 kΩ). Another input was designed for supplying an additional perturbation through resistor R_4_ in view of future impedance measurements but this has not been implemented yet. This scheme is displayed in Fig. 3.

*Standard configuration:* R_1_ = 10 kΩ. R_2_ = 15 kΩ. R_4_ = not connected. R_7_ = 510 Ω, R_8_ = 1 kΩ, R_11_ = 1.5 kΩ. R_12_ = 1 kΩ. R_9_ = R_10_ = 1 kΩ. R_3_ = R_5_ = 10 kΩ. CF_2_ = CF_1_ = 15 pF. CF_3_ = 1 nF. R_6_ = 1 MΩ. This configuration is suitable for probing a 1 mM ferrocene solution in acetonitrile with a 0.5 mm diameter platinum electrode at scan rates between 0.05 and 5 Vs^−1^.

We underline here that Teensy 3.5 analog/digital inputs are 5 V tolerant, giving the possibility to remove resistors R_7_ and R_8_ but this was not tested in the present study.

#### PassStat 2.2: Remote control by Bluetooth for Android smartphone

The teensy card may integrate a remote control via Bluetooth with the RX and TX pins. Here, we chose the HC05 module to establish a serial communication with a smartphone. In this case, an Arduino program should be first uploaded in the Teensy. Then, the Teensy can be disconnected from the computer and powered by a 5 V battery. An application was developed with Android Studio to establish communication. Like for a computer, all parameters can be entered from the phone and the voltammogram is displayed after the acquisition.

*Standard configuration:* identical to PassStat 2.1.

### Analytical formulation for the different configurations

We below develop the analytical formulations for the different potentiostat configurations, considering ideal operational amplifiers. We tried to write this section to be understandable by readers not familiar with electronics. The role of capacitors CF_1_ to CF_3_ that are present to minimize noise and stabilize the system is then neglected. We consider that a tension V_in_ is entered at the potentiostat input. This tension is applied either directly to R_3_ from the Teensy A22 (or A14 for Teensy 3.2) or Analog Discovery outputs (PassStat 1.0 and 2.1) or is obtained by conversion from the Teensy A22 pin tension through OA1.

With ideal amplifiers in linear regime we have:

- E^+^ = E^−^ the voltages are equal at both inputs
- i^+^ = i^−^ = 0 no current flows through the input

In our scheme, the working electrode potential is kept constant at the ground (or deported ground).

## Control of the working electrode potential

### PassStat 1.0

The tension and currents are labeled after the pins of the LMC 6484 component.

In this classical formulation, we have by application of node law at point 6:

(V_in_ – V_6_) /R_6_ = − (V_8_ – V_6_)/R_5_ since I_6_ = 0.

Here V_6_ = V_5_ = V_12_ = V_13_ = 0 (virtual ground).

With R_6_ = R_5_ we have V_8_ = − V_in_ = V_9_ = V_10_ the tension applied to the reference electrode.

Thus V_13_ – V_10_ = E = V_in_ is the working electrode potential.

### Ohmic drop compensation

Because of ohmic losses in solution, the voltage applied to the faradaic impedance is in fact not E but E – R_S_.I, where R_S_ is the solution resistor between the working and reference electrodes. Ohmic drop is particularly problematic with solutions of poor conductivity and/or at relatively large scan rates. Electronic compensation allows to circumvent this problem, with some limitations explained in references [25], [26], [27], [28], [29]. Here positive feedback is operated thanks to variable resistor R_pos_. It was implemented only onto PassStat 1.0 that provides access to large scan rates.

Application of node law on pin 6 leads to:

V_in_/R_3_ + V_14_/R_pos_ + V_10_/R_5_ = 0.

Hence with R_3_ = R_5_.

E = V_13_ – V_10_ = 0 – V_10_ = V_in_ + R_5_V_14_/R_pos_ = V_in_ + (R_6_R_5_/R_pos_).I.

By diminishing R_pos_ the feedback is increased. It reaches 100% compensation for R_6_R_5_/R_pos_ = R_s_ where R_s_ is the solution resistor. In practice, near 100% compensation, an oscillatory behavior appears. Capacitor CF_3_ damps these oscillations, at the expense of a bandwidth reduction. The damped oscillations appear at the potential inversion on Fig. 13 (see below).

**PassStat 2.0**

(V_in_ – V_6_) /R_6_ = −(V_8_ – V_6_)/R_5_ since I_6_ = 0.

Here V_6_ = V_5_ = V_12_ = V_13_ = 1.65 V (deported virtual ground created by voltage divider made with R_9_ and R_10_).

V_8_ = V_9_ = V_10_ = - V_in_R_5_/R_6_ + V_6_(1 + R_5_/R_6_) = - V_in_ + 2 V_6_ with R_5_ = R_6_.

Thus V_13_ – V_10_ = E = V_in_ – V_6_.

Since the Teensy card analog output ranges from 0 to 3.3 V, setting V_6_ to 1.65 V allows to reach electrode potentials between −1.65 and + 1.65 V. The compliance is thus limited.

**PassStat 2.1 and 2.2**

Here, V_2_ = V_3_ = 2 V thanks to the bridge divider made with R_11_ and R_12_.

(V_1_ – V_2_) /R_2_ = - (V_Teensy_ – V_2_)/R_1_ since I_6_ = 0.

Hence V_1_ = V_in_ = V_2_(1 + R_2_/R_1_) – V_Teensy_xR_2_/R_1_.

The analogic output of the Teensy ranges from 0 to + 3.3 V. With R_2_/R_1_ = 1.5 V_1_ = V_in_ ranges from 5 to 0.05 V.

Next.

V_6_ = V_5_ = V_12_ = V_13_ = 2.5 V.

Here V_6_ = V_5_ = V_12_ = V_13_ = 1.65 V (deported virtual ground created by voltage divider made with R_9_ and R_10_).

V_8_ = V_9_ = V_10_ = - V_in_R_5_/R_6_ + V_6_(1 + R_5_/R_6_) = - V_in_ + 2 V_6_ with R_5_ = R_6_.

Thus V_13_ – V_10_ = E = V_in_ – V_6_.

Therefore the accessible electrode potential E = V_13_ – V_10_ ranges from −2.45 to + 2.5 V. The compliance is increased compared to PassStat 2.0.

For the Teensy card, the correct command to send in order to apply the desired potential to the working electrode is calculated in the Python software. Similarly the corresponding currents are directly provided by the program.

**Current reading**

**PassStat 1.0**

Here V_14_ = R_6_.I is the tension directly read by the oscilloscope.

**PassStat 2.0**

Here V_14_ = R_6_.I + V_13_ is the tension directly read by the A0 analog input of the Teensy. V_14_ is limited by the supply voltages thus ranges between 0 and 3.3 V. If R_6_I would be too large or too low, the signal would then simply saturate.

**PassStat 2.1 and 2.2**

V_14_ = R_6_.I + V_13_ as above, but here may range between 0 and 5 V. A0 input should be inferior to 3.3 V otherwise the card would be damaged. Hence, we used an additional voltage divider (resistors R_7_ and R_8_ to limit the maximum tension to 3.3 V.

### Design files

Software and firmware**:** Python and android studio are open source softwares.

## Design files summary

**Table:** 

**Design file name**	**File type**	**Open source license**	**Location of the file**
PassStatDesign.zip	CAD files of the circuit	Creative commons v4	*https://zenodo.org/record/5719382#.YZwJ17rjJhE*
Arduino files	Arduino files to be loaded in the Teensy 3.2 or 3.6 card	GNU GPL v3	*https://zenodo.org/record/5719382#.YZwJ17rjJhE*
Software Python		GNU GPL v3	*https://zenodo.org/record/5719382#.YZwJ17rjJhE*
Software Android		GNU GPL v3	*https://zenodo.org/record/5719382#.YZwJ17rjJhE*
Android app		GNU GPL v3	*https://zenodo.org/record/5719382#.YZwJ17rjJhE*

This design file contains all the necessary files to implement or modify the potentiostat design with the open source software KiCad.

*Bill of materials*

## Bill of materials summary

**Table:** 

**Designator**	**Component**	**Number**	**Cost per unit -currency**	**Total cost – currency**	**Source of materials**	**Material type**
Capacitor 1 µF	C1, C2	2	0.20 €	0.40 €	https://fr.rs-online.com/web/	Electrolytic
Capacitor 47 µF	C3, C4	2	0.20 €	0.40 €	https://fr.rs-online.com/web/	Electrolytic
Capacitor 15 pF	CF1, CF2	2	0.10 €	0.20 €	https://fr.rs-online.com/web/	Ceramic
Capacitor 1 nF	CF3	1	0.10 €	0.10 €	https://fr.rs-online.com/web/	Ceramic
Connector 01x03	J1, J2	2	0.05 €	0.10 €	https://fr.rs-online.com/web/	Metal
Connector 01x03	J3, J4	2	0.05 €	0.10 €	https://fr.rs-online.com/web/	Metal
Jumper	JP1, JP2	2	0.05 €	0.10 €	https://fr.rs-online.com/web/	Metal
Jumper	JP3, JP4	2	0.05 €	0.10 €	https://fr.rs-online.com/web/	Metal
Resistor 10 kΩ	R1, R3, R4, R5	4	0.10 €	0.40 €	https://fr.rs-online.com/web/	Composite
Resistor 15 kΩ	R2	1	0.10 €	0.10 €	https://fr.rs-online.com/web/	Composite
Resistor 1 kΩ	R8, R9, R10, R12	4	0.10 €	0.40 €	https://fr.rs-online.com/web/	Composite
Resistor 1.5 kΩ	R11	1	0.10 €	0.10 €	https://fr.rs-online.com/web/	Composite
Resistor 1 MΩ	R6	1	0.10 €	0.10 €	https://fr.rs-online.com/web/	Composite
Resistor 510 Ω	R7	1	0.10 €	0.10 €	https://fr.rs-online.com/web/	Composite
Quad CMOS Rail-to-Rail Input and Output Operational Amplifier, DIP-14/SOIC-14	U1	1	3.21 €	3.21 €	https://fr.rs-online.com/web/	Semi-conductor
Dual In Line Support	U1	1	0.273 €	0.273 €	https://fr.rs-online.com/web/	Composite
IC Sockets		1 row of 20	0.742 €	0.742 €	https://fr.rs-online.com/web/	Composite
Printed Circuit Board		1	4.30 € (price for 20)	4.30 €	https://www.eurocircuits.com/	Polymer
Teensy 3.2		1	26.39 €	26.39 €	https://www.lextronic.fr/	Hardware
Teensy 3.6		1	39.60 €	39.60 €	https://www.lextronic.fr/	Hardware
Analog Discovery 2		1	294 €	294 €	https://www.lextronic.fr/	Hardware
Bluetooth Module HC05		1	16.90 €	16.90 €	https://www.lextronic.fr/	Hardware
µUSB cable		1	3.00 €	3.00 €	https://www.lextronic.fr/	Cable
External Battery NX 5000 mAh (cheapest models will also work)		1	18.70 €	18.70 €	https://www.1001piles.com/lithium-ion-batterie-externe-universelle-5v-5000mah-103724.html	Hardware

We advise to use DIL support for the quad amplifier but this is not mandatory. IC sockets for R6 and CF_3_ should conversely be used since these components need to be adjusted depending on the electrode size and concentration of electroactive compound to be analyzed.

The cheapest prize with a Teensy 3.2 is 40 €. A system working with Bluetooth module and Teensy 3.6 costs 65€ + price of external battery. For fast scan voltammetry with Analog discovery 2 the price is 305 €, which is still much lower than commercial systems.

## Build instructions

**Fig. 4:**
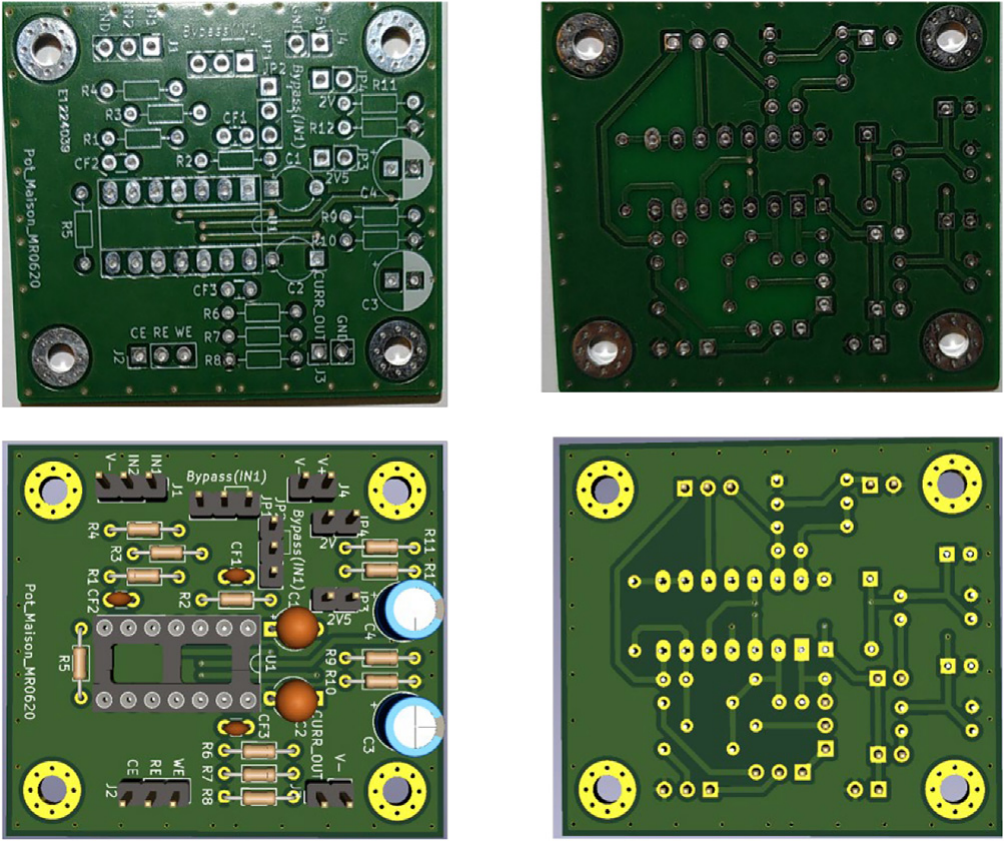
Electronic card used to realize the different configurations and view extracted from KiCAD.

**Fig. 5:**
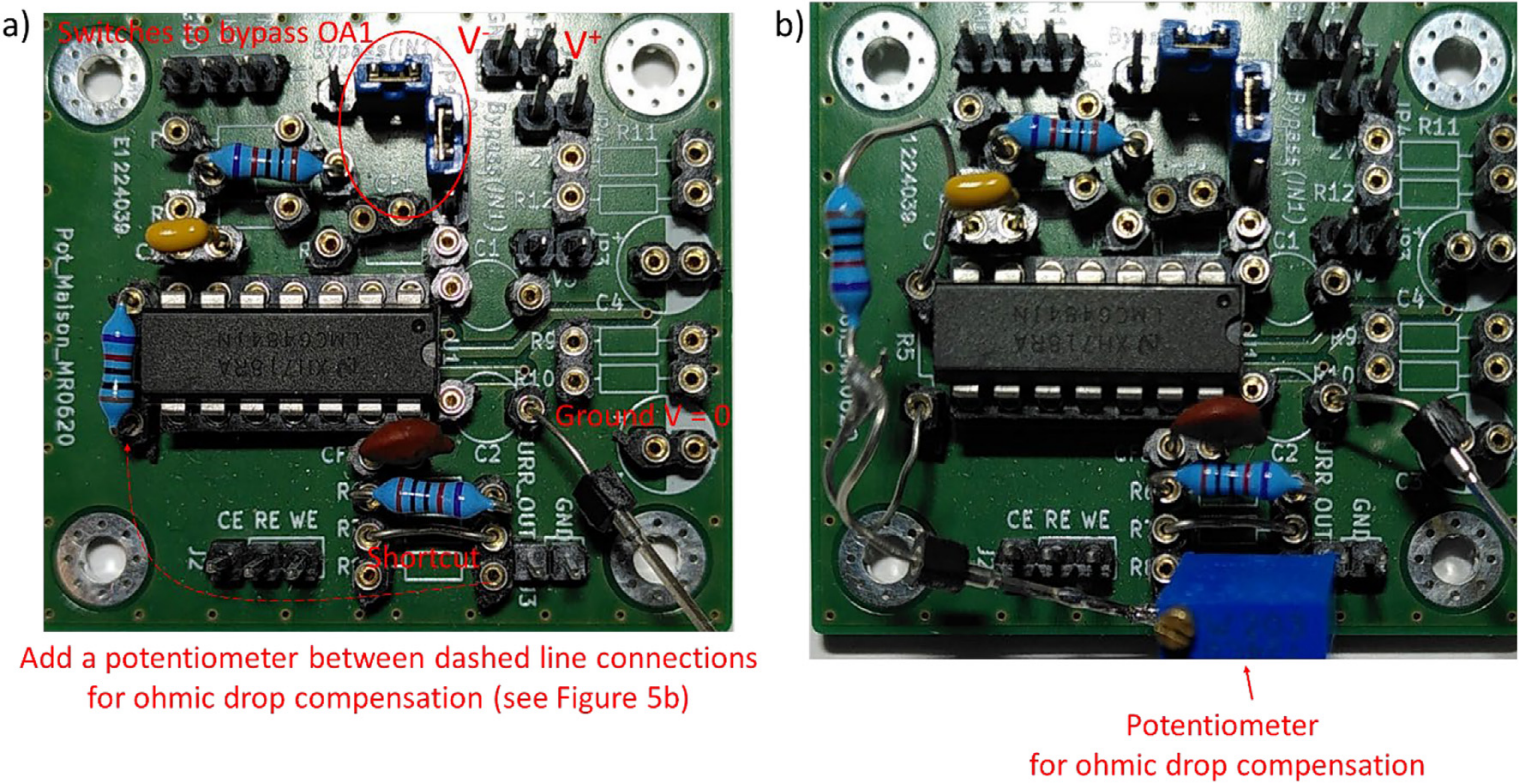
PassStat 1.0 without (a) and with (b) ohmic drop compensation. For (b) a lead was soldered to R_5_ and positive feedback was applied thanks to a 200 kΩ potentiometer (this value should be adapted for different conditions, see calculations above).

**Fig. 6:**
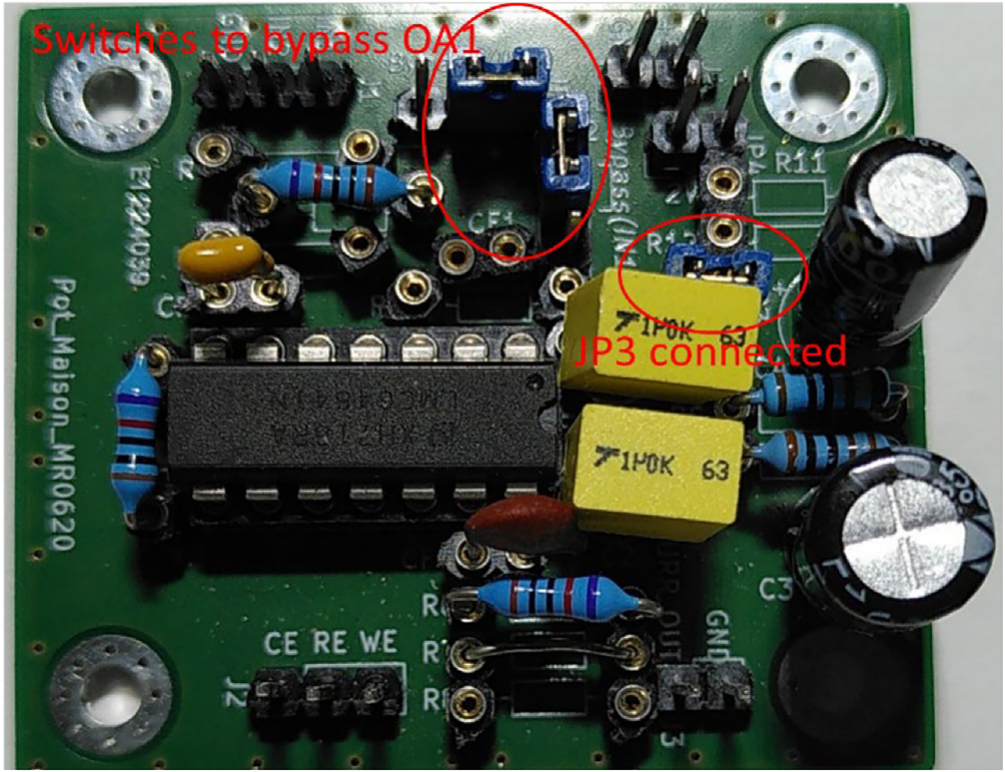
PassStat 2.0. Ground is deported to 1.65 V thanks to resistors R_9_ and R_10_.

**Fig. 7:**
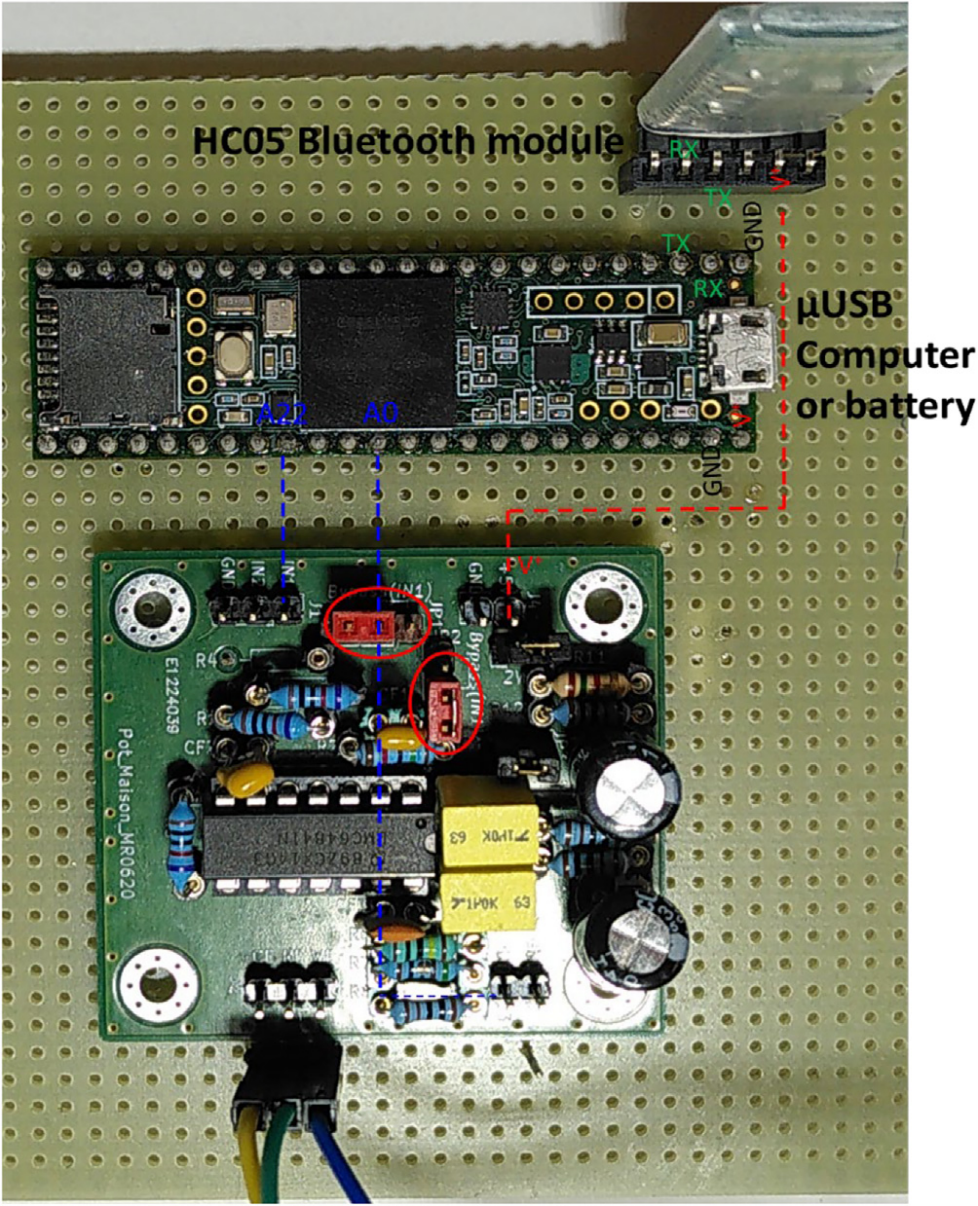
PassStat 2.2 mounted on a PCB. Power is supplied at the µUSB port by a battery to supply the Teensy 3.6 card and the HC05 module. For PassStat 2.1, HC05 is not connected and connection is made directly from a computer.

**Fig. 8:**
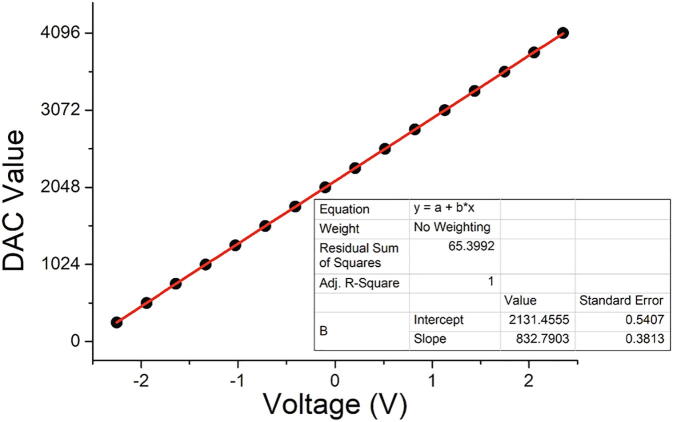
Calibration curve obtained by measuring the tension applied to a 100 kΩ resistor with R_6_ = 100 kΩ. DAC = 2131 correspond to an electrode potential of 0 V. It can be entered directly in the program together with the slope 832.8. 12 bits resolution was used here.

The electronic card is presented in Fig. 4. Jumpers JP1 and JP2 are present to switch between different configurations, and bypass OA1 for PassStat 1.0 and 2.0. They can be replaced by soldered shortcuts if a single configuration is used. Fig. 5, Fig. 6, Fig. 7 present pictures of the different potentiostat implementations (See Fig. 8.).

## Operation instructions

A first program should first be implanted in the Teensy 3.6 card. A computer is necessary for this step. This program contains both CV or SWV methods. When the Teensy is powered, the program waits for parameters arriving on the serial port (methods, potentials, scan rate etc). The orders may be sent either by a computer or by an Android smartphone. For each connection a specific program is provided: CV_SWV.ino for computer and swv_cv_bluetooth.ino for Android Connection with a smartphone is lower (9600 bauds) than with a computer (115200 bauds). The interface was programmed in Python for computer control and with Android Studio for a smartphone.

Should Teensy 3.2 be used instead of Teensy 3.6, A22 should be replaced by A14.

### Computer control

The Python program is organised with three subprograms. swv_cyclique.py should be started by the operator. The port number (that can be read in Arduino program) should be entered prior choosing the method. Then if CV is chosen subprogram volta.py is activated whereas swv.py is activated for SWV. The potentiostat can be controlled from PC under Windows 7 or Windows 10, Macintosh or Linux computers. The port board definition however should be defined in the three python programs and this definition should be adapted for each operating system. We deliver the PC version here. To adapt to Linux or Macintosh, activate the correct line by removing the comment symbol (#) and comment the other ones. We provide below an example for the main swv_cyclique program and for PC:

Line 32:  #PORT_BOARD = “/dev/cu.usbmodem”  # for MAC CPU.

Line 33:  self.PORT_BOARD = “COM”  # for PC CPU.

Line 34:  #self.PORT_BOARD=“/dev/ttyACM”  # for linux.

See lines 23–25 for volta.py and lines 31–33 for swv.py.

We installed Anaconda 3 with Spyder 4 but any Python environment should be operative. Note that the libraries time, math and pyserial should be installed in Python (command pip install pyserial).

### Android smartphone control

The program Potentiostat.apk can be installed on any Android smartphone that operates with Android 4 or superior (ignore security warnings). It was developed with Android Studio that allows to create an application. Datas are saved in android/datas/fr.Achit.lecourantpasse. The code itself is contained in the 4 MainActivity files that interact together. Presentation is handled in the.xml files. The application should be operative on most screens. Should the reader modify the codes, a new app can be generated in the build tab. The first activity handles the choice of method (as above). The second one concerns CV and the third one SWV. The fourth one displays the voltamogram.

### Calibration

All the analytical formulations for the different configurations supposed an USB voltage of 5.00 V, but we observed that the effective USB voltage changes from one computer to another. Such variations induce shift on the positive input of the OAs and then on the applied potential and measured current. This does not prevent to perform experiments, but as for any apparatus a calibration procedure is necessary if accurate measurements are desired. For that, a resistor equal to R_6_ should be used. The specific Arduino Teensy_DAC_ADC program is provided as SI for that purpose.

#### Potential calibration

Here, a correspondence should be established between the digital/analogic scale (from 0 to 4095 for a 12 bits operation) and the applied tension between working and reference electrode (ideally from −2.5 to +2.5 V but in practice a smaller range). For that, DAC values should be sent and the applied tension should be measured with a high impedance voltmeter. A straight line should be observed excepted at the extremities of the potential inversion.

We provide the program Teensy_DAC_ADC that allows to send a DAC value to pin A22 and to enter the tension read by the voltmeter. The line provided in Figure S5 was traced by recording the tension by steps of 250 on the DAC from 250 to 4250. The slope and intercept should be entered in the Python and Android programs as explained below.

#### Current calibration

The DAC value corresponding to the intercept should be entered in the program. Check that the tension applied to the calibration resistor is 0 ± 0.002 V. The current is thus 0. Note the ADC value and enter this value in the line that contains OFFSET_ADC_TEENSY (see below).

To check the calibration procedure, run a CV between −1 and +1 V on a resistor equal to R_6_. A symmetric straight line should be observed.

This procedure is applicable for 12 bits or 16 bits operation (in this case 4095 should be replaced by 65535).

The following values should be changed in the volta.py and swv.py programs.

self.QUANT_DAC_TEENSY = 1./832.79  # extracted from calibration process.

  self.OFFSET_DAC_TEENSY = 2131.45 # extracted from calibration process.

  self.QUANT_ADC_TEENSY = 3.3/4095.

  self.OFFSET_ADC_TEENSY = 2070.0.

  self.COEFF_CONV_TEENSY = 1.51.

The following values should be changed in the Android studio first three activities:

final double QUANT_DAC_TEENSY=(1/832.79);
final double gain = −1;
final double OFFSET_DAC_TEENSY = 2131.45;
final double OFFSET_ADC_TEENSY = 2070.0;

## Validation and characterization

Since PassStat 1.0 works with an arbitrary function generator and an oscilloscope, all potentiostatic techniques may be implemented. For PassStat 2, the present software version includes cyclic voltammetry and square wave voltammetry. Additional techniques will be implemented in the future.

### Materials and methods

The counter electrodes were platinum wires. The reference electrode was either a home-made AgCl/Ag electrode, or a platinum wire (for low currents or high speed). The home-made working electrodes were either a 0.25 mm radius Pt disk (standard conditions), a 9B pencil lead from Cretacolor (paracetamol), a 2 µm radius Pt disk (low currents) or a 15,200 µm^2^ gold ball.

Ferrocene (Aldrich), tetrabutylammonium hexafluorophosphate (Alfa Aesar), Citric acid (Aldrich) and acetonitrile (VWR) were used withour further purification. Doliprane 1000 mg tablets from Sanofi were used to prepare a 1 mM paracetamol solution with 0.100 M citric acid as supporting electrolyte.

Analog Discovery 2 from Digilent was used for PassStat 1.0.

The Teensy 3.6 acquisition card and HC05 Bluetooth module were purchased from Lextronic.

LMC 6484 operational amplifier and other electronic components were all purchased from radiospares.

The circuit was designed with KiCad 5.1.0, an open source software for electronic design. The circuit may also be implemented onto test breadboards.

### Test on a dummy cell

**Fig. 9:**
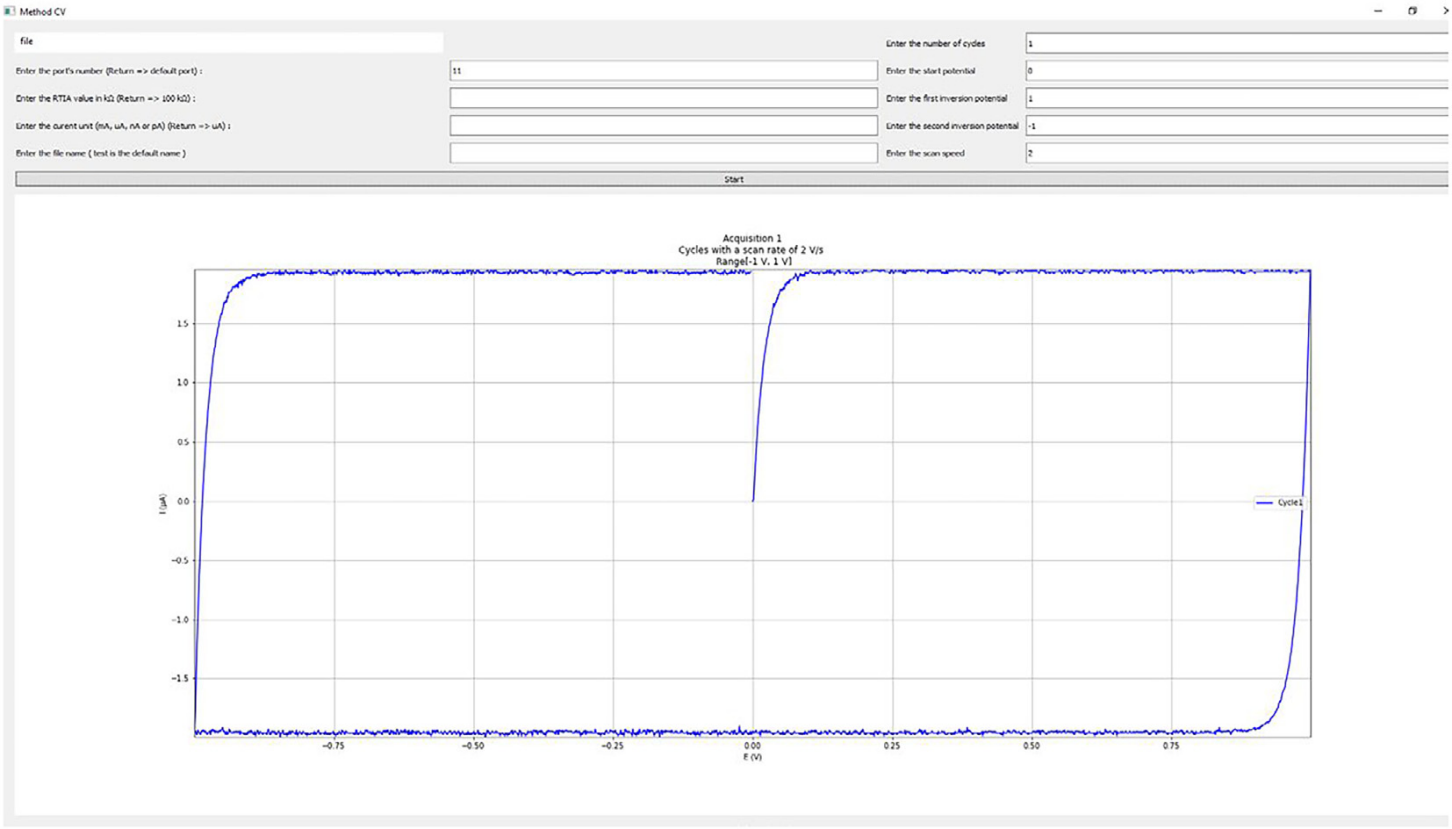
Voltammogram obtained at 2 Vs^−1^ for a dummy cell with R = 10 kΩ and C = 1 µF. Screenshot of the software window.

To probe the electronic system prior to perform real electrochemical experiments or to identify a problem in case of failure, it may be useful to use a test circuit called a dummy cell. Here we are using R = 10 kΩ in series with C = 1µF and a scan rate of 2 Vs^−1^. Counter and reference electrodes are connected at the same place (shortcircuit). At the potential start or inversion, exponential variations are observed up to plateaus for which the capacitive current is: i_c_ = Cv as represented in Fig. 9.

### Classical electrochemical conditions.

**Fig. 10:**
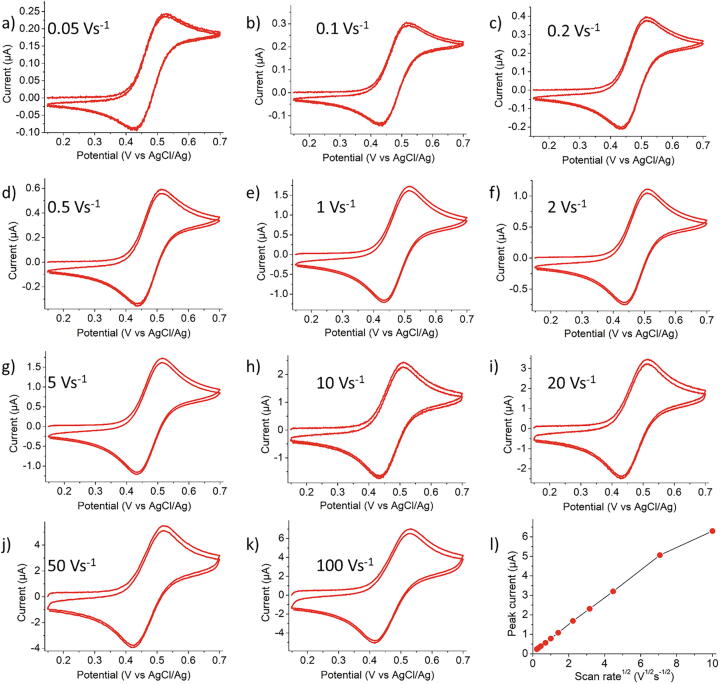
A–k) Cyclic voltammogram obtained at different scan rates for a 0.25 mm radius Pt electrode in acetonitrile containing 1 mM ferrocene and 0.10 M TBAPF_6_ with PassStat 2.1. l) Peak potential versus scan rate.

Fig. 10 presents cyclic voltammograms acquired in the range 0.05–100 Vs^−1^ using a 0.25 mm radius platinum electrode with PassStat 2.1 for a 1 mM ferrocene solution in acetonitrile, in presence of 0.10 M tetrabutylammonium hexafluorophosphate (TBAPF_6_) as supporting electrolyte. CF_3_ was 1 nF in all cases. R_6_ was 1 MΩ from 0.05 to 5 Vs^−1^ and 100 kΩ above 5 Vs^−1^. Plotting peak current against ν^1/2^ displays a good linearity as expected for purely diffusive behavior (see Fig. 10l). A deviation is however observed at 100 Vs^−1^ because at this scan rate the signal is altered both by ohmic drop effects and by the too high value of the R_6_CF_3_ low pass filter value (0.1 ms with R_6_ = 100 kΩ and CF_3_ = 1 nF, see section 3.3 for optimization at important ν).

**Fig. 11:**
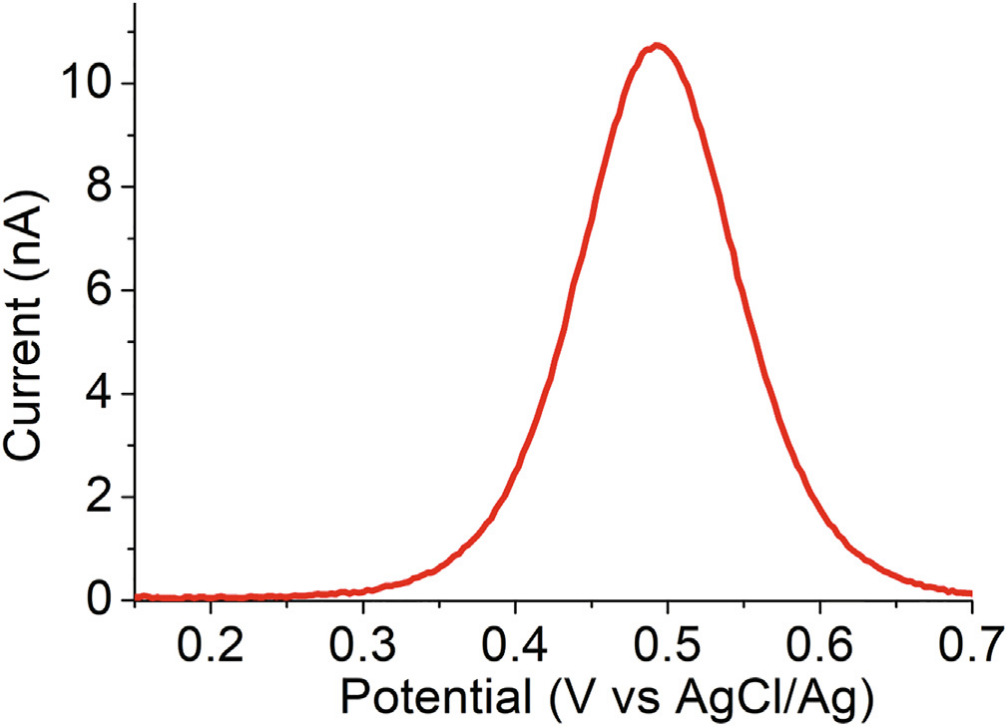
Square wave voltammogram obtained at 0.1 Vs^−1^ for a 0.25 mm radius Pt electrode in acetonitrile containing 1 mM ferrocene and 0.10 M TBAPF_6_ with PassStat 2.1. R_6_ = 1MΩ and CF_3_ = 1 nF.

In the same conditions, a square wave voltammogram was acquired at an equivalent scan rate of 0.1 Vs^−1^, potential steps of 20 mV and potential increment of 3 mV (see Fig. 11). A very smooth and well-resolved curve is observed, validating the potential of the device towards analytical purposes.

**Fig. 12:**
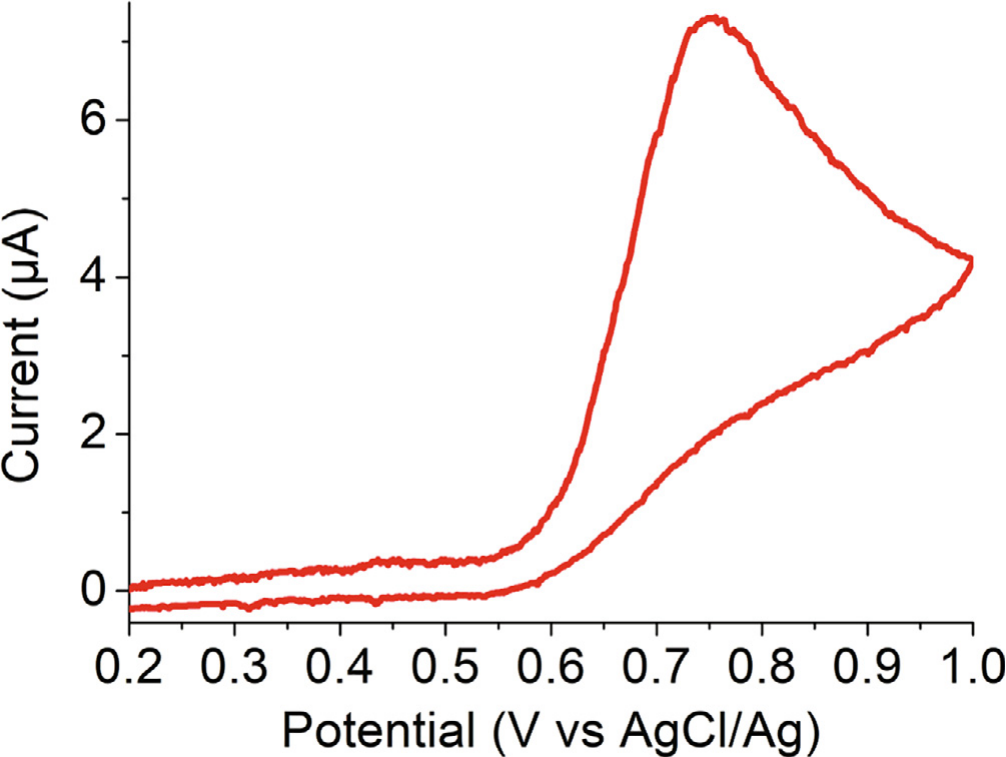
Cyclic voltammogram obtained on a 9B pencil lead at 0.1 Vs^−1^ in an aqueous solution containing 0.10 M citric acid as supporting electrolyte and 1 mM paracetamol originating from a pharmaceutical tablet with PassStat 2.2 (smartphone application). R_6_ 100 kΩ, CF_3_ = 1 nF.

In addition, to demonstrate the efficiency of the device for low cost experiments towards identification of potential fake drug analysis, we analyzed an aqueous solution prepared from a paracetamol tablet with a 9B pencil lead as working electrode. Various pencil lead electrodes have indeed been proposed as cheap electrode material in view of electroanalysis [33], [34], [35]. Here, paracetamol concentration was 1 mM and we choose 0.10 M citric acid as supporting electrolyte. Many different conditions are described in the literature [15], [16], [17], [18], [19], [20], [21], [22], [23], [24], and we found that an acid medium provided a better reproducibility. Moreover, citric acid can be purchased easily so that preparing this electrolyte may be easier than other classical buffers in non-ideal experimental conditions. This experiment was performed thanks to remote control with a smartphone. Fig. 12 confirms that no additional noise is added in these conditions.

**Fig. 13:**
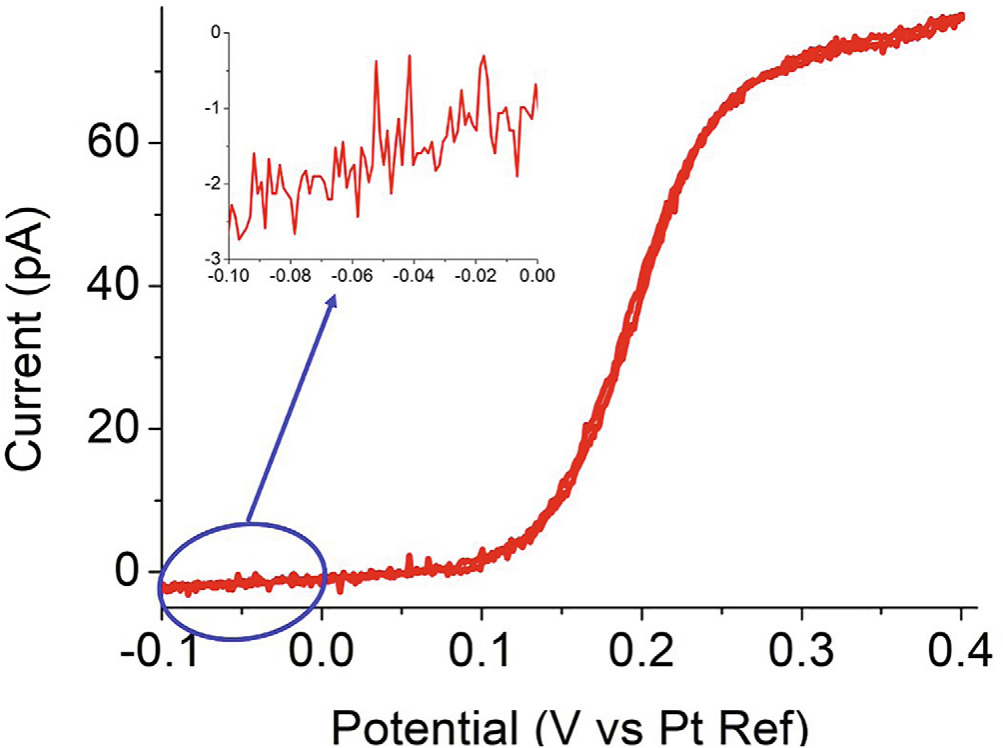
Cyclic voltammogram obtained with a 2 µm radius Pt ultramicroelectrode in an acetonitrile solution containing 25 µM of ferrocene and 2.5 mM tetrabutylammonium hexafluorophosphate at 10 mVs^−1^ with PassStat 2.1. R_6_ = 1 GΩ, CF_3_ = 100 pF.

### Detecting low currents

When using ultramicroelectrodes (UMEs), *i.e.* electrodes of micrometric or nanometric dimensions, the CV shifts from transient to steady state at low scan rates. Ultramicroelectrodes are useful to increase signal/noise ratio in analytical chemistry. Additionally, UMEs allow to work with low amounts of supporting electrolyte [28]. Hence, we resorted here onto a solution containing only 25 µM of ferrocene and 2.5 mM of TBAPF_6_ in CH_3_CN. Since very low currents are then concerned, resistor R_6_ should be increased to 1 GΩ here. Fig. 13 presents the cyclic voltammogram obtained at 0.01 Vs^−1^ for a 2 µm radius platinum electrode with the PassStat 2.1 configuration for which R_6_ was set to 1 GΩ and CF_3_ to 100 pF. A current plateau of only 72 pA is observed. In these conditions, the RMS noise level was found to be less than 0.5 pA as shown in the inset of Fig. 13. The current quantification step with these parameters is only 0.075 pA with a 16 bits resolution, a value only slightly slower than the noise level. This second experiment demonstrates that even if in most of the case a 12 bits resolution is sufficient, this is not the case in such extreme conditions.

Finally, we underline that here R_6_CF_3_ = 100 ms so that the temporal resolution is still excellent. CF_3_ may be further diminished if dynamic events such as impacts of biological exocytotic release should be caught. There is moreover still room for optimization, notably by using PassStat 2.0 configuration and/or oversampling the data.

### Fast scan voltammetry

**Fig. 14:**
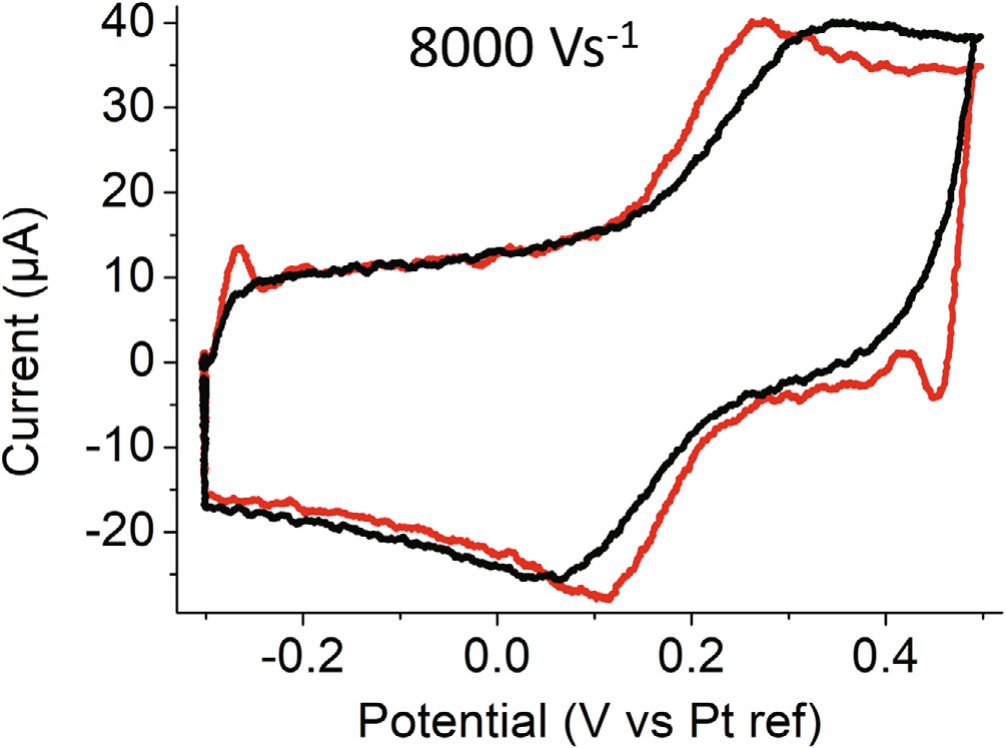
Cyclic voltammogram obtained with a gold ball ultramicroelectrode in an acetonitrile solution containing 1 mM of ferrocene and 0.10 M tetrabutylammonium hexafluorophosphate at 8000 Vs^−1^ without (black) and with (red) ohmic drop compensation. R_6_ = 10 kΩ, CF_3_ = 3.3 pF. (For interpretation of the references to colour in this figure legend, the reader is referred to the web version of this article.)

Another interesting property of UMEs is to give access to low time scales (nanoseconds in the best conditions) [32]. We evaluated the PassStat 1.0 configuration in the fast scan range with a gold ball ultramicroelectrode produced by melting a 12.5 µm radius gold wire. The electrode area was estimated to be 15200 µm^2^ in a calibration procedure. In the black CV of Fig. 14, realized for a 1 mM solution of ferrocene in acetonitrile containing 0.10 M TBAPF_6_, the capacitive plateau is not attained immediately because of ohmic drop within the electrochemical cell at 8000 Vs^−1^. Even if UMEs drastically reduced ohmic losses, the large current densities pertaining to large ν finally alter the signal. To face this problem, ohmic drop compensation was applied by adding a positive feedback with the potentiometer R_Pos_ displayed in red in Fig. 5b. At 100% compensation, *i.e.* when the equivalent feedback resistor equals the solution one, the red curve shows oscillations at the limits of the potential ramp. The current peaks are then no more distorted by the ohmic drop, as long as positive feedback occurs quickly enough. Our previous reports explain in detail interactions between electrode size, electrolyte composition and electronic set-up so as to carry out ohmic drop compensation with a maximum accuracy.

### Conclusion and perspectives

We presented above several possible schemes to build a low cost but powerful potentiostat for analytical electrochemistry. The electronic board is easy to implement and repair, and can be realized at a low cost. The simplicity of the scheme stems from the use of a quad amplifier component. The python and android interfaces are fully open source. Only a minimal training is necessary to use this potentiostat, and electrochemical performances are close to those of commercial potentiostats so that it could is suitable for measurements at a research level [36]. Should one need to adapt the design to specific needs additional elements could be easily implemented. Those could be independent power supplies precisely regulated to apply voltages independent from the USB or battery ones, additional filters and amplification stages or specific faster amplifiers [11], [31]. Future implementations could add a bipotentiostat control for measurements with two working electrodes and electrochemical impedance spectroscopy [37], [38]. The Analog Discovery 2 or Teensy card are furthermore useful to add further possibilities, for example control of a rotating disk electrode or couple electrochemistry with other techniques such as spectroscopic ones or pH sensing for example [39]. We invite the reader to check software and hardware updates. The present work is thus the first stone to develop other electrochemical applications for example analytical measurements on the ground [40].

## CRediT authorship contribution statement

**Mélicia Caux:** Investigation. **Anis Achit:** Software. **Kethsovann Var:** Investigation. **Gabriel Boitel-Aullen:** Investigation. **Daniel Rose:** Conceptualization. **Agnès Aubouy:** review and editing. **Sylvain Argentieri:** Review and editing. **Raymond Campagnolo:** Conceptualization. **Emmanuel Maisonhaute:** Conceptualization, Supervision, writing, review and editing.

## Declaration of Competing Interest

The authors declare that they have no known competing financial interests or personal relationships that could have appeared to influence the work reported in this paper.
